# PCR inhibition in qPCR, dPCR and MPS—mechanisms and solutions

**DOI:** 10.1007/s00216-020-02490-2

**Published:** 2020-02-12

**Authors:** Maja Sidstedt, Peter Rådström, Johannes Hedman

**Affiliations:** 1 Swedish National Forensic Centre, Swedish Police Authority, 581 94 Linköping, Sweden; 2 Applied Microbiology, Department of Chemistry, Lund University, P.O. Box 124, 221 00 Lund, Sweden

**Keywords:** Blood, Digital PCR, DNA polymerase, Humic substances, Massively parallel sequencing, PCR inhibitors

## Abstract

DNA analysis has seen an incredible development in terms of instrumentation, assays and applications over the last years. Massively parallel sequencing (MPS) and digital PCR are now broadly applied in research and diagnostics, and quantitative PCR is used for more and more practises. All these techniques are based on in vitro DNA polymerization and fluorescence measurements. A major limitation for successful analysis is the various sample-related substances that interfere with the analysis, i.e. PCR inhibitors. PCR inhibition affects library preparation in MPS analysis and skews quantification in qPCR, and some inhibitors have been found to quench the fluorescence of the applied fluorophores. Here, we provide a deeper understanding of mechanisms of specific PCR inhibitors and how these impact specific analytical techniques. This background knowledge is necessary in order to take full advantage of modern DNA analysis techniques, specifically for analysis of samples with low amounts of template and high amounts of background material. The classical solution to handle PCR inhibition is to purify or dilute DNA extracts, which leads to DNA loss. Applying inhibitor-tolerant DNA polymerases, either single enzymes or blends, provides a more straightforward and powerful solution. This review includes mechanisms of specific PCR inhibitors as well as solutions to the inhibition problem in relation to cutting-edge DNA analysis.

## Introduction

DNA polymerase–based analysis has become indispensable in many societal functions. Established techniques such as conventional PCR and real-time quantitative PCR (qPCR), as well as emerging ones such as digital PCR (dPCR) and massively parallel sequencing (MPS), are key tools for decision-making in, for example, forensic DNA analysis, food safety, clinical diagnostics and bioterrorism preparedness [1–4]. One of the main analytical challenges is that the samples of interest often contain low amounts of nucleic acids in combination with a challenging matrix [5, 6]. Molecules from the sample matrix, target cells or reagents added during sample preparation that affect the in vitro DNA polymerization or the fluorescence signal are collectively called PCR inhibitors. To overcome analytical limitations in presence of PCR inhibitors, it is useful to understand the underlying molecular inhibition mechanisms. This critical review gives an overview of PCR inhibition mechanisms in relation to qPCR, dPCR and MPS, and solutions to overcome inhibition problems are proposed. Knowledge is collected from the fields of forensics, clinical diagnostics, food safety and veterinary medicine.

**Fig. 1:**
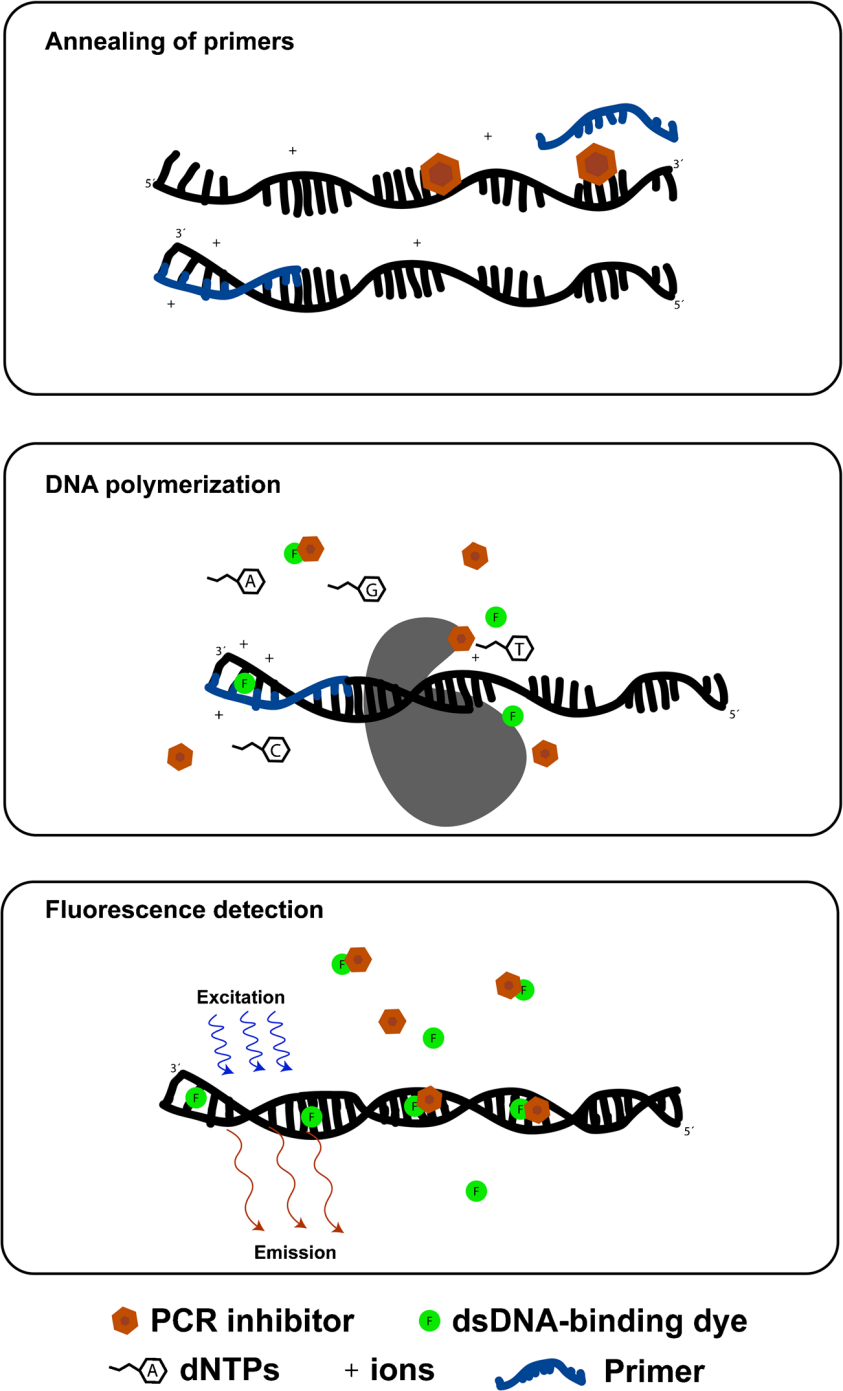
Illustration of the critical subreactions in PCR. PCR inhibitors may affect any of these subreactions, i.e. disturb annealing of primers, affect DNA polymerase activity or impair fluorescence detection

The strength of PCR is that it enables amplification and detection of specific nucleotide sequences from one or just a few target molecules. Efficient in vitro DNA polymerization demands high DNA polymerase activity as well as favourable interactions between nucleic acids (target denaturation and primer annealing), meaning that both biochemical and biophysical processes are involved [7, 8]. Any compound affecting any of the critical reagents or the subreactions in the polymerization process thus acts as an inhibitor (Fig. 1).

Fluorescence measurement is the main means to detect and quantify nucleic acids in DNA polymerase–based analysis, thanks to the ease-of-use and excellent limits of detection [9, 10]. Fluorophores attached to primers or nucleotides are essential in, for example, forensic STR analysis [11], Sanger sequencing [12] and sequencing-by-synthesis MPS [13, 14]. Fluorescence intensity is also used to monitor amplicon generation in qPCR and dPCR. An often overlooked disadvantage of fluorescence-based analysis is that any substance that disturbs the function of the fluorophore, for example by fluorescence quenching, will impair the analysis [15, 16]. Fluorescence quenching can be caused through different mechanisms, for example collisional quenching and static quenching [17]. In collisional quenching, the quenching molecule comes into contact with the excited-state fluorophore, and in static quenching, the quencher forms a non-fluorescent complex with the fluorophore in its ground state.

Digital PCR analysis has been proven to be less affected by PCR inhibitors than qPCR [18–20]. The main reason for the more accurate dPCR quantification in presence of PCR inhibitors is that end-point measurements are applied, meaning that there is no reliance on amplification kinetics. For qPCR, on the other hand, quantification cycle (Cq) values are linked to a standard curve and any inhibition effect skewing the Cq values will directly affect quantification. However, complete inhibition has been shown to occur at lower amounts of humic acid in qPCR compared with dPCR, indicating that the increased PCR inhibitor tolerance in dPCR quantification cannot be explained by the use of end-point measurements alone [16]. Possibly, the partitioning of the samples into many minute reactions play a role in the elevated resistance to inhibitors due to less interaction between inhibitor molecules and the molecules involved in the polymerization process. Still, considerable differences in dPCR quantification accuracy in presence of PCR inhibitors have been shown for different DNA polymerases [16].

Around 2011, the first benchtop platforms for MPS were released. This simplified DNA sequencing, for example enabling cost-effective and fast analysis of bacterial genomes in outbreak events [21, 22]. Today, MPS is increasingly applied in, for example, clinical diagnostics and forensics [23, 24]. MPS has opened up for many new applications in forensic DNA analysis, such as phenotype prediction, enhanced mixture analysis and body fluid identification [25, 26]. MPS analysis relies on in vitro DNA polymerization and also on fluorescence measurements when considering the sequencing-by-synthesis technology, one of the most commonly used sequencing approaches [27].

qPCR, dPCR and MPS are all vulnerable to molecules that interfere with the DNA polymerase or the nucleic acids in the reactions. Apart from the actual amplicon generation, the detection of amplicons through fluorescence measurements must function optimally. There are several molecules that can interfere with the PCR, e.g. through lowering of the DNA polymerase activity, by interacting with nucleic acids or by quenching fluorescence. It has been reported that different PCR assays, including different primer and target sequences, may be affected differently by inhibitors [28, 29]. Also, larger fragments are in general more difficult to amplify in the presence of PCR inhibitors than smaller ones. It is crucial to understand and control the impact of PCR inhibitors on the quality and reliability of PCR and MPS data, specifically concerning impure samples with low amounts of DNA. A comprehensive list of relevant PCR-inhibitory molecules can be found in [30].

## Challenging samples

**Fig. 2:**
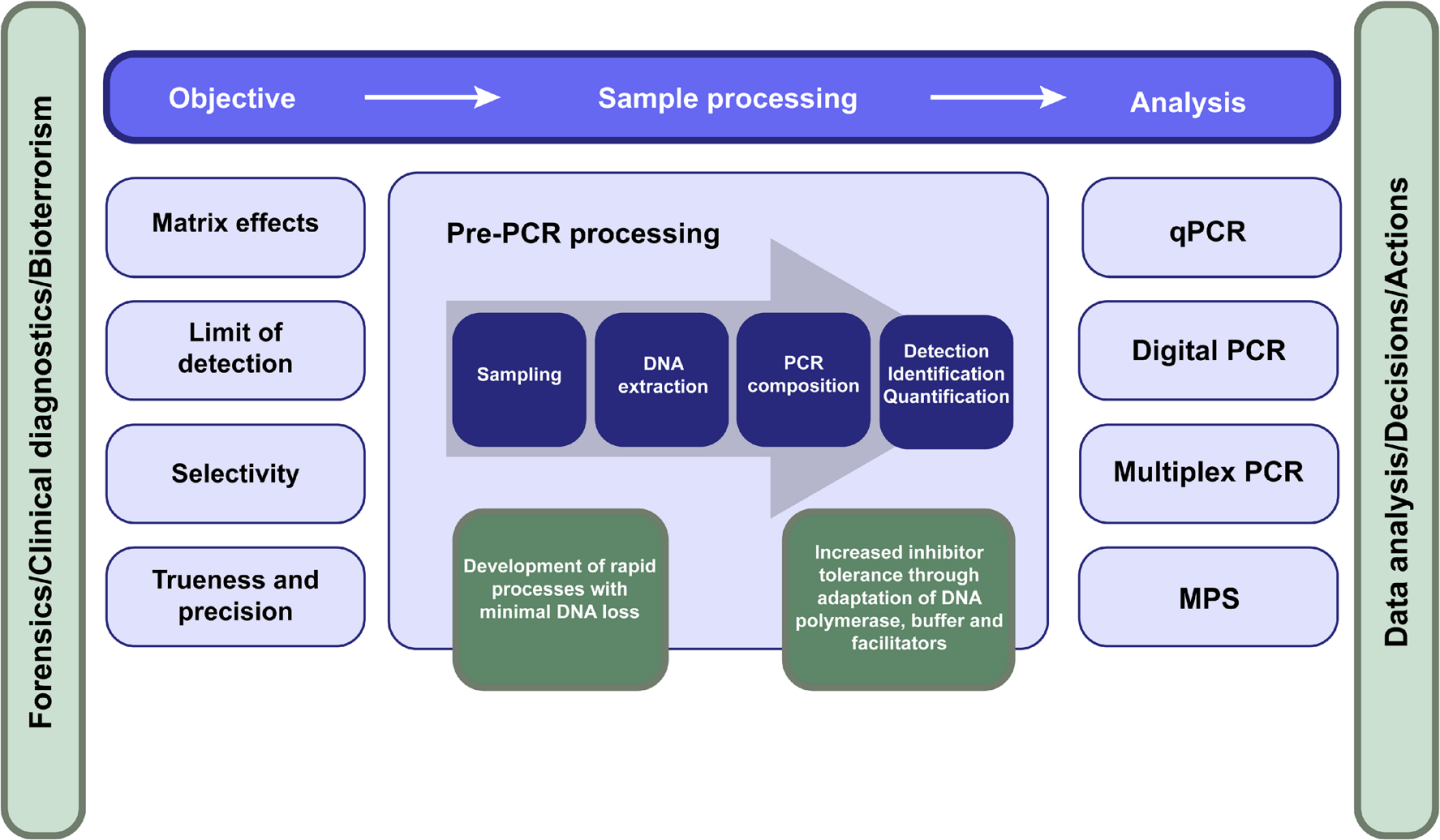
The general workflow for PCR-based analysis. To ensure optimal analytical success for challenging samples, important performance characteristics to investigate in method validation are matrix effects, limit of detection, selectivity and trueness and precision. In the pre-PCR processing concept, the different parts of the analytical process are viewed as links in a chain. Thus, to overcome limitations with low DNA amounts or PCR inhibitors, one of the most vital steps is to apply a DNA polymerase-buffer system that is compatible with the sample matrix

The general PCR workflow consists of sampling, extraction of the nucleic acids and thereafter detection, quantification or identification of specific nucleic acids (Fig. 2). PCR may be applied to accurately detect low DNA amounts, to quantify the amount of a certain microbial pathogen or to determine the identity of the person that left DNA at a crime scene. The objective will guide the important sampling step. It is vital that the sample is representative of the material tested and that uptake and release of cells is optimized [30, 31]. Further, sampling should aim to minimize the uptake of PCR inhibitors from the sample matrices while maximizing the target uptake [30]. There are different strategies for sampling, where swabbing with a cotton or nylon swab is commonly applied in forensics and microbial testing [32–34].

The sample preparation serves to generate homogeneous DNA extracts from heterogeneous samples using, for example, biochemical or physical principles [35]. DNA is released from the cells by DNA extraction, and if needed, the extracts are further purified. A common method for DNA extraction is based on Chelex resin, providing a convenient and quick method with low cost [36]. Cationic magnetic beads and silica-based filters provide efficient purification of nucleic acids and have been applied in many automated methods [37]. Other advanced DNA purification methods include subjecting the DNA extract to high pressure and focusing DNA in a small area on a gel [38, 39]. In recent years, there has also been a focus on developing integrated microfluidic systems for sample preparation and amplification of nucleic acids [40]. The drawback of extensive purification is that it leads to substantial DNA loss, generally with recovery rates from 10 to 80% [41, 42]. The opposite of extensive purification is direct PCR methods, where the sample preparation step is minimized or left out entirely [43]. Here, the advantage is that the DNA loss is avoided, but one disadvantage is that high amounts of PCR inhibitors may be present. A direct PCR approach used in forensic DNA analysis cut the DNA profiling time from 10–12 h to 2–3 h by applying the inhibitor-tolerant DNA polymerase Phusion Flash [44]. The methodology was most effective for samples with high DNA amounts, where a sub-sample of controlled size could be added to the PCR.

Many different types of challenging samples may be relevant in bioterrorism preparedness, food safety, archaeogenetics and forensics. Any type of human tissue deposited on any surface (e.g. cigarettes, fabrics, outdoors [45]) may be of interest in forensic DNA analysis. In microbial testing of food, the sample matrix may be, for example, raspberries or minced meat. These various sample types cause analytical complications linked to PCR-inhibitory molecules from the target cells/tissues, the sample matrix and/or reagents added during sample processing.

When a crime has been committed outdoors, there may be a need to analyse human DNA from soil samples. Trained search dogs are capable of locating small amounts of body fluids not visible to the naked eye. The challenge is to isolate and analyse these small amounts of human cells in a complex background of plant or soil material [43]. In environmental studies, soil and sediment are often the source of genetic material, for example when studying the microbiota or tracing pathogens. Soil has a high content of humic substances that are degradation products of lignin decomposition [46]. Humic substances can be divided into three categories: humin which is black and insoluble, humic acid that is dark brown and soluble at neutral or alkaline pH and fulvic acid which is yellowish and soluble in water at all pH levels [47]. Humic and fulvic acids are two heterogeneous groups of dibasic weak acids with carboxyl and hydroxyl groups. Statistical calculations indicate that in 1 kg of humic acid, there are not two molecules that are identical [48, 49]. Humic acids are generally larger than fulvic acids which in turn contain more oxygen and less carbon than humic acid [47, 50, 51]. Humic acid molecules have molecular weights of up to approximately 100,000 Da compared with about 10,000 Da for fulvic acid [46]. Humic acid has been identified as the main PCR inhibitor in sample matrices of soil and sediment [52–54].

Blood is often analysed for nucleic acids both in clinical diagnostics and in forensic DNA analysis. Blood is composed of plasma (approximately 55% of total blood volume), white blood cells and red blood cells (approximately 45% of total blood volume) [55]. Potential PCR inhibitors that have been identified in blood are immunoglobulin G, lactoferrin, haemoglobin and anticoagulants such as EDTA and heparin [56–58]. In clinical diagnostics, there is a need for quick and reliable analysis by means of direct PCR analysis of blood samples [59]. In forensic DNA analysis, it is not uncommon that a recovered blood stain has been exposed to tough environmental conditions, posing harsher analytical challenges due to low DNA levels and DNA degradation. Thus, to avoid the need for extensive purification where DNA is lost, it is vital to control and circumvent the PCR inhibition effects.

## Inhibition of DNA polymerization

In vitro DNA polymerization may be inhibited by molecules that have a direct negative effect on the polymerase activity, e.g. through binding to or degrading the enzyme, or molecules that affect the ion content or bind to nucleic acids, thus hindering primer extension [60]. To design a DNA polymerase-buffer system that is compatible with the sample matrix, there is a need to understand how different molecules impact the polymerization.

### DNA polymerase inhibitors

Haemoglobin and haematin have been reported to cause inhibition of the DNA polymerization in qPCR and dPCR [58, 61]. More recent work has shown that haemoglobin and haematin cause lowered DNA polymerase activity which impacts the amplification efficiency [62]. The release of iron trichloride from haemoglobin has previously been suggested to be responsible for the PCR inhibition [57], but recent findings indicate another main mechanism of inhibition since iron trichloride by itself showed different effects compared with haematin and haemoglobin [62]. Further, the principal inhibitory effect of haemoglobin has been suggested to be caused by the haem groups, and therefore, haematin (a derivative of haem) is often used as a model for blood in validation studies [63–65]. Each molecule of haemoglobin contains four haematin molecules. Thus, four times more haematin should give the same effects as a certain amount of haemoglobin if the main inhibitory effect is caused by haem alone. However, haemoglobin has been shown to be a more potent inhibitor than haematin since 470 μM haemoglobin or 3000 μM haematin was needed to cause complete amplification inhibition in dPCR analysis [62]. This indicates that it is not solely the haem group that is responsible for the haemoglobin-induced inhibition.

Humic substances, specifically humic acid and fulvic acid, have long been acknowledged to cause PCR inhibition [54, 66]. DNA polymerization inhibition has been suggested to occur through binding to DNA or by directly impacting the DNA polymerase activity [53, 67, 68]. However, humic acid does not bind to DNA under regular PCR conditions [15, 52]. The level of PCR inhibition has been shown to be similar for various soils and standardized humic acid preparations, and it has been suggested that phenolic structures of humic substances are likely responsible for the DNA polymerase inhibition [52]. Humic acid has been used in several studies as a defined PCR inhibitor representing soil and sediment [15, 52, 69].

**Fig. 3:**
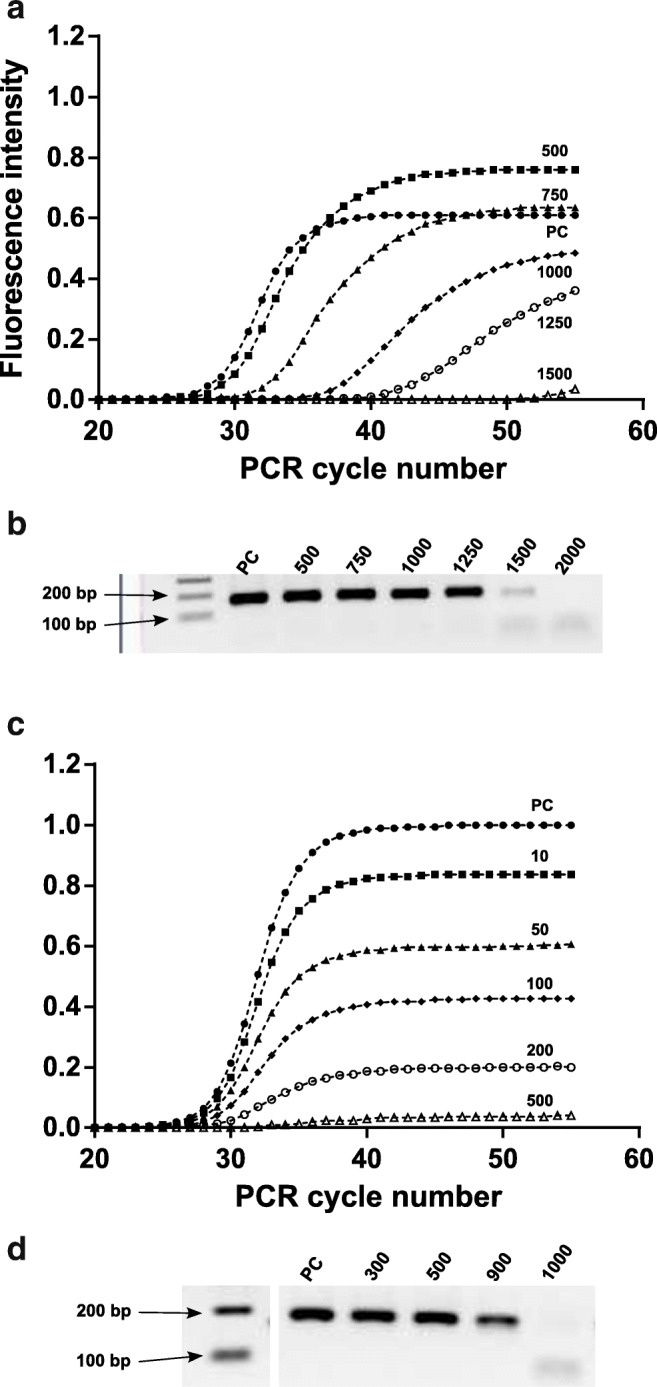
Examples of how PCR inhibition may be manifested in qPCR. **a** Amplification curves for analysis with increasing amounts of fulvic acid that inhibits amplification. **b** Corresponding agarose gel electrophoresis results for the amplification in **a**. **c** Amplification curves for analysis with increasing amounts of humic acid that quenches the fluorescence. **d** Corresponding agarose gel electrophoresis results for the reactions in **c**. The numbers depict the amounts of humic acid or fulvic acid spiked into the reactions. PC (positive control) denotes a reaction where water was added instead of PCR inhibitor. EvaGreen dye was used for detection. Reprinted from Analytical Biochemistry, 487, pp. 30–37, Title: Humic substances cause fluorescence inhibition in real-time polymerase chain reaction, Authors: Maja Sidstedt, Linda Jansson, Elin Nilsson, Laila Noppa, Mats Forsman, Peter Rådström and Johannes Hedman. Copyright (2015), with permission from Elsevier [15]

When investigating PCR inhibition mechanisms, it is vital to apply various DNA polymerases and buffer systems. Otherwise, the found mechanism may not be relevant in practical settings. A DNA polymerase-buffer system with high tolerance to humic substances (Immolase with 10 μg BSA) was used when studying inhibition mechanisms [15]. There, both humic acid and fulvic acid caused polymerization inhibition, although higher amounts of fulvic acid were needed to impair amplification. An example of how the amplification inhibition effect of fulvic acids is manifested in qPCR may be found in Fig. 3 a and b. Increasing amounts of DNA template or Mg^2+^ did not improve the tolerance to humic acid. Therefore, it was concluded that the amplification inhibition is caused by a direct effect on the DNA polymerase, rather than on template DNA or through chelation of vital ions. High amounts of DNA polymerase can be used as an expensive means to elevate PCR inhibitor tolerance [68], and also to study inhibition mechanisms. Increasing amounts of DNA polymerase (from 1 to 5 U) were applied to support the conclusion that the inhibition mechanism for humic acid, haemoglobin and haematin is a direct effect on the DNA polymerase activity (Table 1) [15, 16, 62]. Bile salts and phytic acid in faeces have also been reported to act as DNA polymerase inhibitors with a direct effect on the DNA polymerase [70, 71]. Phenols introduced during DNA purification can act as inhibitors by denaturation of the polymerase [72]. Additionally, moist snuff tobacco has been reported to have a negative effect on the polymerase activity [73].

**Table 1:** A summary of the PCR inhibition mechanisms for the main PCR inhibitors in soil and blood

Source	Molecule	Effect on DNA polymerization	Effect on fluorescence detection	Proposed mechanism
Soil and sediment	Humic acid	Decreased amplification efficiency, eventually leading to complete amplification inhibition	Quenching of fluorescence (e.g. EvaGreen, SYBR Green I, ResoLight, ROX)	Binds to fluorescent dyes, causing static fluorescence quenching Lowers the activity of the DNA polymerase, likely with the greatest effect in the early cycles of PCR
	Fulvic acid	Decreased amplification efficiency, although less potent than humic acid	Quenching of fluoresence at high concentration. No noted effect in qPCR due to a stronger negative effect on DNA polymerization	Lowers the activity of the DNA polymerase
Blood	IgG	Increased Cq values eventually leading to complete amplification inhibition	No effect	Binds to genomic ssDNA, thereby hindering primer annealing, thus disturbing the initiation of amplification in the first few PCR cycles
	Haemoglobin	Decreased amplification efficiency, eventually leading to complete amplification inhibition	Quenching of fluorescence (e.g. EvaGreen, ROX)	Binds to fluorescent dyes, causing static fluorescence quenching Lowers the activity of the DNA polymerase throughout the PCR
	Haematin	Similar to haemoglobin, although a weaker effect	Similar to haemoglobin, although a weaker effect	Similar to haemoglobin, although a weaker effect

MPS analysis relies on in vitro DNA polymerization and fluorescence measurements for detection. For targeted MPS, where the sequence of interest is amplified prior to sequencing, the targeted PCR amplification is crucial for accurate analysis [74–77]. It is also important to consider the sampling strategy, DNA extraction method and quantity of the DNA, as well as contamination control in the practical workflow of MPS [78]. For pure template samples, it has been shown that the PCR step in MPS contributes to bias in obtained sequences for templates with extreme base compositions [74]. Further, there are reports highlighting the importance of the choice of DNA polymerase to obtain accurate sequencing data [75, 79–81]. For example, when four different genomes with varying GC-contents were amplified with 24 different DNA polymerase buffer systems and one PCR-free protocol, it was found that the best performing enzyme was KAPA HiFi and not the more commonly used Phusion DNA polymerase [81]. It has been observed that PCR inhibitors limit MPS applications [69, 78, 82]. For example, it was noted that humic acid and haematin caused complete amplification inhibition at certain levels in MPS-based analysis with a commercial forensic kit [69]. For MPS methods to be widely used in forensic and clinical routine analysis, there is a need to ensure compatibility with challenging sample types such as blood and soil.

The impact of DNA polymerization errors is a much discussed topic in MPS-based analysis [74, 75, 83–85]. Recently, the effect of humic acid and haematin on MPS-based analysis was investigated [86]. The inhibitors did not impact the nucleotide sequence, but rather the amount of amplicons that was generated in the initial targeted multiplex PCR. Larger amplicons were most severely affected, suggesting that humic acid and haematin impact the DNA polymerase activity in the initial PCR. Further, there was uneven amplification of the different targets in the multiplex PCR. In contrast, low DNA amounts in the initial PCR resulted in evenly lowered read numbers for all markers, which further strengthened the conclusion that the inhibitor effect was on the DNA polymerase and not on DNA [86].

### Nucleic acid inhibitors

Immunoglobulin G (IgG) has previously been suggested to inhibit polymerization by binding to single-stranded genomic DNA (ssDNA), hence interfering with primer annealing [56]. This was recently confirmed using alternative methods to study the different subreactions of PCR [62]. The main effect of IgG on dPCR was elevated and more dispersed Cq values. In qPCR, the slopes of the amplification curves were not affected by IgG, but delayed amplification (higher Cq values) was observed. This implies inhibition due to DNA binding. The electrophoretic mobility shift assay (EMSA) is a method used to study protein-DNA interactions. EMSA experiments showed that IgG preferentially interacts with single-stranded high-molecular weight genomic DNA [62]. The specific effect on ssDNA was further corroborated in dPCR, where IgG had a substantially stronger inhibitory effect when using ssDNA as template, compared with dsDNA. The interactions between antibodies and DNA have also been studied in other fields, and it has been shown that a small portion of IgG in serum of healthy people binds to ssDNA [87]. Further, researchers studying lupus monoclonal antibodies showed that there are different binding mechanisms of antibodies to DNA, where some preferentially bind to dsDNA in complex with a DNA polymerase [88]. Another molecule that has been identified as a nucleic acid inhibitor is collagen in bone, causing inhibition by interaction with template DNA which affects the DNA polymerase processivity [67, 89]. Cellulose in cigarette filter papers or wood and bilirubin in faeces are other compounds that have been implicated to interfere with the DNA polymerization through binding to DNA [73, 90, 91].

Isothermal titration calorimetry (ITC) is a technique for thermodynamic and kinetic studies, which relies on measuring the heat released or absorbed during a chemical reaction. ITC has been used to determine the interactions between the DNA polymerase and the primer-template complex as well as the salt dependence of binding for pure samples [92, 93]. For example, the investigation of the thermodynamics for the DNA-binding characteristics revealed that *Taq* DNA polymerase binds efficiently to the primer-template complex over a range of temperatures, with the highest affinity at 40 to 50 °C [92]. ITC has also been applied to study the DNA polymerization, independent of fluorophores and primer annealing efficiency, showing that haematin, but not IgG, inhibits the DNA polymerase activity [62]. Calorimetry is a promising methodology to study the different subreactions in PCR and thus investigate specific PCR inhibition mechanisms.

### Inhibition of fluorescence detection

**Fig. 4:**
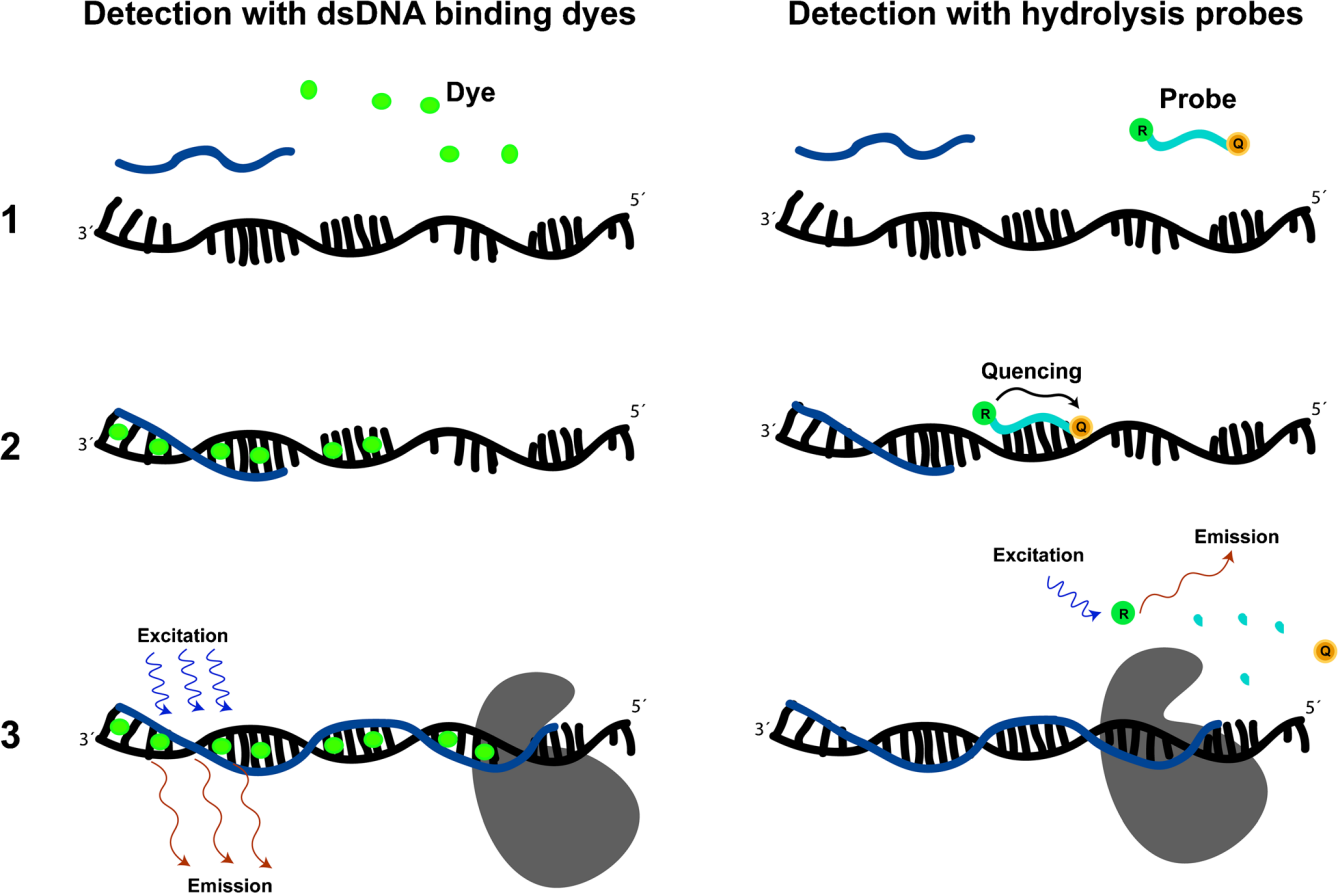
Schematic representation of the two most commonly used fluorescence detection systems in PCR-based applications

The phenomenon of Förster resonance energy transfer (FRET) is used in the design of dual-labelled hydrolysis probes for detection of specific amplicons in qPCR and dPCR (Fig. 4) [94–96]. Probes for qPCR are labelled with a fluorescent dye acting as reporter, e.g. 6-carboxyfluorescein (FAM), and a second fluorescent dye serving as quencher, e.g. 6-carboxy-tetramethyl-rhodamine (TAMRA). The fluorophores are attached to an oligonucleotide, and as long as they are in close proximity, TAMRA will quench FAM fluorescence. The application of these oligonucleotide probes relies on annealing of the probe to the target sequence, and subsequent hydrolysis of the phosphodiester bonds in the probe by the 5′-3′ exonuclease activity of the DNA polymerase. When the probe is cleaved, the reporter fluorescence will no longer be quenched due to increased distance between the reporter and the quencher molecule. Probes differ from dsDNA-binding dyes in that the fluorescence signal is directly connected to the amplification of the specific target sequence.

Cyanine dyes are a class of fluorescent dyes with high affinity for binding to DNA. They have proven to be very useful in qPCR due to their characteristic increased fluorescence upon binding to dsDNA (Fig. 4) [97–99]. There are two modes of non-covalent dye interaction with DNA: intercalation and surface binding. Surface binding can occur either within the major groove, which is common for larger molecules such as proteins, or within the minor groove. DNA-binding dyes generally intercalate or bind to the minor groove. Molecules that bind to DNA through intercalation are often cationic with planar aromatic rings, whereas minor groove binders usually have more flexible structures. The binding of dye molecules to DNA is the key to monitor the generation of amplicons during amplification. However, the dyes should not have too high binding affinity for DNA since this can hinder amplification [100].

The first reported qPCR applications used ethidium bromide to monitor the increase in amplicon amount [101]. Not long after, SYBR Green I was applied for the same purpose [102] and SYBR Green I is still the most commonly used cyanine dye in PCR applications. SYBR Green I has been proposed to function through intercalation in combination with minor groove binding via interaction through the positively charged amino group of the elongated arm [103–105]. It has also been observed that SYBR Green I exhibits sequence-specific binding, with preferential binding to amplicons with high GC-content [106]. SYBR Green I inhibits PCR at moderate concentrations due to its strong binding affinity for dsDNA (*K*_d_ 3.1 nM reported in [103]), elevating the melting temperature of the DNA double helix up to 10 °C [100]. Apart from inhibiting polymerization, the high binding affinity for DNA leads to the generation of more primer-dimer products for SYBR Green I than for the dye SYTO-82 [100]. Alternative dyes such as EvaGreen, SYTO-9 or SYTO-82 have been shown to have a much lower affinity for dsDNA compared with SYBR Green I, which may explain why these are less PCR inhibitory [100, 107, 108]. EvaGreen has been reported to have an intrinsic affinity constant of 3.6 × 10^5^ M^−1^ and the two acridine orange moieties of EvaGreen likely cooperatively intercalate into dsDNA [109].

### Fluorescence quenching mechanisms

The introduction of qPCR using fluorescence detection has brought great benefits to the field. However, any molecule that interferes with the fluorophores may inhibit the detection of amplicons. This inhibition may be due to quenching of the fluorescence or by hindering a dye from binding to DNA. Changes in the ion content can also impact DNA-binding characteristics of both cyanine dyes and hydrolysis probes [99]. Further, if an inhibitor has a negative effect on the 5′-3′ exonuclease activity, this could lead to inhibition since the DNA polymerase would not be able to hydrolyse the probe [110]. Fluorescence inhibition can lead to an underestimation of the DNA quantity or even false negative results which in turn could have dramatic effects on the decisions made based on the outcome. Still, research related to fluorescence inhibition has been scarce.

Humic substances have previously been shown to cause a negative effect on non-PCR-based fluorometric methods used to quantify nucleic acids [49, 111]. In PCR-based studies, both lake sediment and humic acid caused systematically lowered qPCR fluorescence signals when the DNA-binding dye EvaGreen was used [15]. The results showed lowered qPCR end-point fluorescence intensity or failed detection, although clear bands of the correct PCR product were detected in agarose gel electrophoresis. An example of how fluorescence inhibition caused by humic acids may be manifested in qPCR is provided in Fig. 3 c and d. When studying the effects of humic acid on other dsDNA-binding dyes, i.e. ResoLight, SYBR Green I and SYTO-82, it was revealed that these were all affected by fluorescence quenching, although the degree of inhibition differed [15]. For example, EvaGreen fluorescence was relatively more quenched than SYTO-82, and no apparent correlation between the quenching effect and dye concentration was seen. Likely, the difference in sensitivity to fluorescence quenching between the dyes is explained by their different structures and binding affinities to both DNA and humic acid. Using hydrolysis probes for detection in presence of humic acid revealed no negative effect on the fluorescence signals, i.e. the amplification curves and the product detected with gel electrophoresis were in concordance [15]. Indigo dyes have also been implicated to disturb fluorescence detection by causing background fluorescence [67].

Fluorescence spectroscopy has been used to investigate the underlying mechanisms of the fluorescence quenching caused by humic substances [15]. There, the EvaGreen signal was lowered by increasing amounts of both humic acid and fulvic acid. This was apparent for free EvaGreen dye molecules as well as for EvaGreen bound to DNA. Fulvic acid caused no fluorescence inhibition in qPCR. However, fluorescence spectroscopy measurements showed that large amounts of fulvic acid quenched the fluorescence of qPCR dyes. This was not observed in qPCR because such high amounts of fulvic acid inhibited polymerization, thus masking the quenching effect. There were no observed effects on the fluorescence emission spectra by humic acid or fulvic acid. Stern-Volmer plots were used to investigate the nature of the fluorescence quenching [15]. A linear relationship was shown, indicating that static quenching is the major quenching mechanism. In summary, humic acid causes static quenching of dsDNA-binding dyes as confirmed with qPCR and fluorescence spectroscopy measurements.

In work dedicated to determine the PCR inhibition mechanisms of molecules in blood, it was identified that haemoglobin, and to a lesser extent haematin, also quenches the fluorescence of DNA-binding dyes [62]. Haemoglobin has previously been suggested to quench fluorescence in non-PCR applications and fluorescein has been found to bind within the central cavity of haemoglobin [112, 113]. However, no systematic fluorescence quenching of the hydrolysis probe reporter dye (FAM, a derivative of fluorescein) was observed using qPCR or dPCR measurements with humic acid or haemoglobin. The fact that humic acid does not quench the hydrolysis probe fluorescence may be due to that this fluorophore is bound to an oligonucleotide and not free in solution. Other reasons for the difference in quenching between probes and DNA-binding dyes may be binding affinities or the ionic charge, as cyanine dyes generally have a positive charge and humic acid and fluorescein generally are negatively charged in qPCR (pH around 8.3).

Bovine serum albumin (BSA) is a potent facilitator reducing the polymerization-inhibitory effects of, for example, humic substances [15, 91]. However, BSA did not counteract the fluorescence quenching from humic acid [15]. For example, fluorescence quenching of EvaGreen was observed at similar levels with 2 and 10 μg BSA in the reactions. This may be due to a higher binding affinity between the dyes and humic acid, compared with BSA and humic acid. However, when there is no dye present, humic acid interacts with BSA rather than with the DNA polymerase as witnessed by the increased amplification inhibitor tolerance with BSA [15].

High amounts of humic acids are tolerated in dPCR, but result in elevated normalized fluorescence of the amplification curves [16]. The normalized fluorescence is the signal of the FAM fluorophore normalized to the passive reference ROX. Examination of the fluorescence intensity revealed that 375–750 pg/nL humic acid quenched the ROX fluorescence to approximately half of the intensity compared with reactions without humic acid. This could cause analytical problems since the fluorescence threshold applied to distinguish positive and negative reactions may be inaccurate. When analysing reactions with whole blood in dPCR, 5% blood resulted in quenching of the ROX fluorescence to less than half the intensity compared with reactions without blood [62]. The same effects were observed with haemoglobin present in the reactions, in both qPCR and dPCR. In summary, both humic acid and haemoglobin inhibit the fluorescence signal for several free dyes, e.g. SYBR Green I, EvaGreen and ROX reference dye [15, 16, 62] (Table 1). Dithiothreitol (DTT), used in several DNA extraction protocols, has also been observed to act as a fluorescence quencher, causing lowered fluorescence intensity of the passive reference dye MustangPurple in qPCR [114].

## Solutions to PCR inhibition

### Monitoring PCR inhibition

In order to avoid false negative and incorrect results in routine diagnostics, it is important to monitor and control PCR inhibition. This entails applying proper quality assurance and quality control measures [115, 116]. Method validation is a prerequisite prior to implementation to ensure that the methods perform well for the samples of interest [117]. Apart from the general performance characteristics that should be investigated in a validation study (e.g. limit of detection, selectivity, trueness and precision), PCR inhibition should be properly addressed (Fig. 2) [118, 119]. It is also crucial to include relevant sample types and matrices in the validation study, i.e. matrices expected to be present in the analysis. For this purpose, reference material mimicking commonly encountered matrices may be prepared and applied [120]. The possible outcome of such an experiment, where DNA is analysed with qPCR in the presence of increasing amounts of inhibitors, is provided in Fig. 3. Apart from showing that inhibition is present, it may be elucidated whether the inhibitors affect the DNA polymerization (Fig. 3a, b) or the fluorescence signals (Fig. 3c, d). This then directs any need for further improving the method. In routine analysis, there are two main ways to assess if the analysis is affected by PCR inhibition: (1) using internal amplification controls (IAC) or (2) investigating amplification kinetics through the actual amplification curves of the target DNA (kinetic outlier detection (KOD)) [30, 121]. IAC is well-established and highly recommended in diagnostics. KOD has not yet been broadly introduced in routine analysis, but may be applied as a complement to IAC. IAC is based on the addition of a non-target DNA fragment at known concentration that is co-amplified with the target, which helps to avoid false negatives [122]. The design of the internal amplification control should enable detection of PCR inhibition, meaning that if the target amplification is affected by inhibition, this can be elucidated from the IAC. For targeted MPS, the initial PCR is most vulnerable to PCR inhibitors [86]. After library preparation, the samples are generally diluted substantially, lowering the risk of inhibitors impacting the sequencing. As a quality control measure, PCR inhibition effects may be monitored prior to sequencing by checking quality and quantity of the generated libraries applying CE-based fragment analysis, e.g. Fragment Analyzer, or qPCR [86, 123].

### Overcoming polymerization inhibition

Today there are a wide variety of commercial DNA polymerases tailor-made for different applications [124]. A critical part of setting up an efficient DNA analysis process is to apply a DNA polymerase-buffer system that is compatible with the sample matrices analysed, thus ensuring optimal limits of detection. The use of alternative DNA polymerases and buffer systems can efficiently circumvent PCR inhibition for challenging samples. A methodology called pre-PCR processing has been proposed to overcome limitations caused by inhibitors and to achieve optimal detection limits by using an appropriate PCR composition [5, 6] (Fig. 2). The aim of pre-PCR processing is to achieve a fast and simple analysis process with minimal loss of nucleic acids [5]. For example, in efforts to improve detection of waterborne RNA viruses, pre-PCR processing was successfully applied to elevate the virus recovery and make the RT-qPCR step more efficient [125]. Further, to achieve higher tolerance to PCR inhibitors, blends of complementary or synergistic DNA polymerases may be applied [73].

The first study that investigated the PCR inhibitor tolerance of different DNA polymerases showed that the widely used Ampli*Taq* Gold and *Taq* DNA polymerases were considerably less resistant to blood (completely inhibited by 0.004% (vol/vol)) compared with DNA polymerases isolated from *Thermus thermophilus* (r*Tth*) and *Thermus flavus* (*Tfl*). The latter were able to amplify DNA without reduced efficiency in the presence of 20% (vol/vol) blood [126]. A screening of 16 DNA polymerases and buffer systems has been performed to find the most robust reaction composition for soil and sediment samples [15]. The four best performing DNA polymerase-buffer systems in that screening were Immolase, KAPA3G, PerfeCta Toughmix and TEMPase. The combination of Immolase and a high amount of BSA (10 μg) proved to be the most inhibitor-tolerant variant, enabling amplification in presence of up to three times more humic substances than the other three [15]. In another study, great differences in tolerance to soil-derived humic substances were observed for six DNA polymerase-buffer systems [52]. In forensic DNA profiling, alternative DNA polymerases were shown to perform better than Ampli*Taq* Gold for various types of crime scene traces [127]. A blend of the two complementary DNA polymerases PicoMaxx HF and Ex*Taq* HS further improved performance [73] and led to a substantial increase in number of usable DNA profiles for both blood and saliva samples when implemented in casework [128]. These studies exemplify the importance of selecting a robust DNA polymerase-buffer system as a means for reaching optimal analytical success for challenging samples.

In efforts to generate new variants of DNA polymerases with elevated inhibitor tolerance, some knowledge of polymerization inhibition has been gained. *Taq* DNA polymerases have been generated by site-directed mutagenesis resulting in greater resistance to inhibitors in blood and soil as well as an increased tolerance to high concentrations of DNA-binding dyes [129, 130]. The authors speculate that the elevated resistance to inhibitors may be due to an altered enzyme speed or improved interaction with the DNA template. More recent efforts have used compartmentalized self-replication [131] or fusion strategies [132] for the design of new DNA polymerases. For example, mutants of *Taq* DNA polymerase that exhibited a higher affinity for primer-template (*K*_d_ 1.1 nM for mutant versus 102 nM for wild-type *Taq*) showed increased tolerance to blood with successful amplification in up to 45% whole blood [131]. By fusing a DNA polymerase to a ssDNA-binding domain, the inhibitor tolerance was improved for human blood, lactoferrin and heparin [133, 134]. In other studies, compartmentalized self-replication has been used to generate new enzymes that showed higher tolerance to humic acid and other inhibitors [135, 136]. One of those enzymes, called 2D9 [135], showed improved abilities to function in the presence of humic substances, but there was no clear explanation of the underlying mechanisms. Further, new enzymes with higher resistance to salts have been generated through a strategy of adding on new protein domains to DNA polymerases [137].

The buffer composition can also be altered to improve polymerization in presence of inhibitors, e.g. applying an elevated pH [138]. Further, changing the ion content or adding various facilitators can enable a higher resistance to PCR inhibitors [15, 130, 139]. BSA is an abundant blood plasma transport protein that binds efficiently to a range of molecules and has been shown to make DNA polymerization more efficient in presence of many inhibitory molecules [52, 91]. A range of BSA amounts have been applied for this purpose, with 0.2 to 20 μg in the reactions [6, 15]. Trehalose is a solute that has been shown to improve the thermal stability of enzymes, a mechanism believed to explain its inhibition-relieving properties, when 2–30% (w/v) is added to the reactions [30, 130, 140].

### Overcoming fluorescence inhibition

The risk for detection inhibition should be considered when analysing challenging samples such as those containing soil or blood. There are several studies where SYBR Green I detection has been applied for analysis of matrices that contain blood or humic acid [141–143], which may have skewed the results and conclusions. Using hydrolysis probes for detection is a much better alternative than dsDNA-binding dyes in order to avoid fluorescence inhibition when humic acid or haemoglobin is present in the reactions. However, for some applications, it may still be preferable to apply dyes. Then, blending of qPCR dyes has been suggested to optimize detection in the presence of molecules that quench fluorescence [144]. Blending of dyes resulted in elevated fluorescence intensities, thus enabling detection of amplicons in the presence of, for example, humic acid. High concentrations of individual dyes inhibit amplification, but a blend of the four dyes EvaGreen, ResoLight, SYBR Green I and SYTO-9 showed higher fluorescence intensities in both presence and absence of humic acid with no or low negative effect on amplification. It is also important to consider that the dye used as a passive reference may be sensitive to fluorescence quenching and that different dyes may be affected differently, such as ROX and MustangPurple [16, 114]. Further, the use of an internal amplification control could help in monitoring samples for fluorescence inhibition as well as polymerization inhibition.

## Conclusions

Depending on the mechanism of the relevant inhibitor, and whether it mainly affects DNA polymerization or fluorescence detection, different strategies may be applied to overcome PCR inhibition. For challenging matrices such as soil and blood, a lot is known about the PCR inhibition mechanisms (Table 1). This knowledge about PCR inhibition mechanisms may be applied to design more robust analysis systems.

A critical part in setting up an efficient DNA analysis process is to apply an inhibitor-tolerant DNA polymerase-buffer system that is compatible with the relevant sample matrices. Selecting alternative DNA polymerases and buffer systems, e.g. through screening, can efficiently circumvent PCR inhibition from challenging samples, which is the core of the pre-PCR processing strategy. When analysing samples containing, e.g., humic acids or haemoglobin, simply applying hydrolysis probes instead of dsDNA-binding dyes can enable a more robust analysis and avoid issues with fluorescence quenching [15]. Further, dPCR may be used instead of qPCR for challenging samples since this technique is not as sensitive to PCR inhibition due to that end-point measurements are used [1].

An increased awareness of the effects that PCR inhibitors can have on the analysis is crucial to ensure that reliable results are generated. Measures for troubleshooting include the assessment of relevant sample types and matrices in method validation [117], as well as implementation of quality control measures such as IACs. For many molecules, the mechanism of inhibition has not been elucidated, i.e. whether they affect the DNA polymerase activity, ion content or nucleic acids. Additionally, the exact mechanisms of DNA polymerase inhibitors, e.g. how certain molecules bind to the polymerase, have not been explained. To achieve optimal analytical success for DNA polymerase–based methods, more studies are needed that investigate the underlying PCR inhibition mechanisms.
